# Photocatalytic Analysis of Titanium Semiconductors Anodized in H_3_po_4_, Hf and *Psidium guajava*-Based Electrolyte

**DOI:** 10.1021/acsomega.5c08657

**Published:** 2026-01-22

**Authors:** Sara Einsfeld Altenhofen, Sandra Raquel Kunst, Luana Góes Soares, Isadora Schell Frozza, Carlos Leonardo Pandolfo Carone, Claudia Trindade Oliveira

**Affiliations:** 125098 Feevale University (ICCT), RS 239, 2755, Bairro Vila Nova, CEP,Novo Hamburgo-RS 93352-000, Brazil

## Abstract

Titanium is a versatile material that can be used both as a semiconductor in heterogeneous photocatalysis and in the medical field, mainly to produce implants aimed at restoring, replacing, and correcting biological structures with poor osseointegration performance. This versatility is directly related to its unique properties, such as low toxicity, chemical stability, ability to absorb UV light, durability, stability at different pHs, and photosensitivity. Therefore, this study aims to compare titanium anodizing in *Psidium guajava* using an anodizing bench with the industrial process. For this purpose, samples anodized in *Psidium guajava* and anodized by the industrial process (H_3_PO4 or H_3_PO_4_ + HF) were compared regarding morphology, roughness, color, electrochemical corrosion tests, and Raman spectroscopy. Additionally, heterogeneous photocatalysis tests were also performed to assess the photocatalytic activity of TiO_2_ in the degradation of methylene blue dye. It was found that the samples anodized with the *Psidium guajava* electrolyte showed high photoactivity (99%) in the degradation of methylene blue, in addition to a performance similar to that of H_3_PO_4_ + HF and better than that of H_3_PO_4_. This can be attributed to the presence of phenolic compounds such as quercetin and indicates that *Psidium guajava* anodizing is an efficient and sustainable alternative to the industrial process.

## Graphical abstract

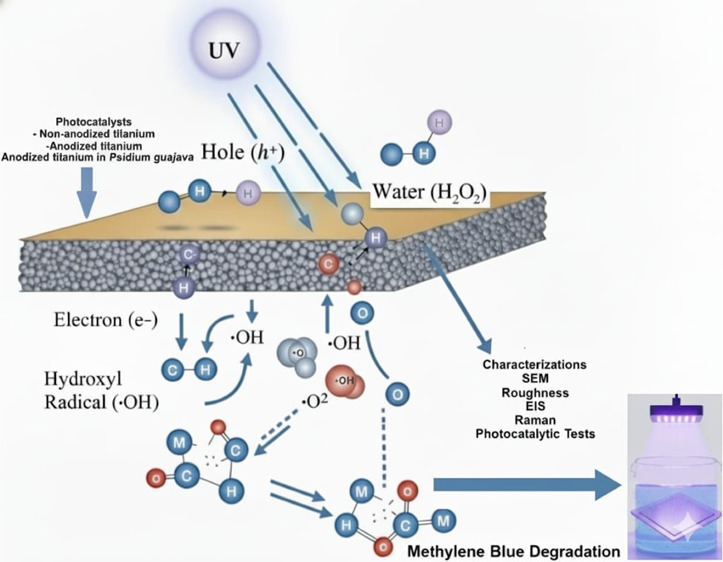

## 1 Introduction

The properties of a metallic biomaterial must be evaluated to avoid rejection in the human body, mainly due to the location of the implant and the medical history of the patient, which can influence the performance of the biomaterial. Among its most relevant properties are photocatalytic activity, biocompatibility, corrosion resistance, mechanical features, osseointegration, wear resistance and processability.^1^

Biomaterials assist in the osseointegration process due to their interaction with the surrounding tissue as if they were a natural tissue, which results in the successful response of the body to implants. Hydroxyapatite [Ca_10_(PO_4_)­6­(OH)_2_] is a type of calcium phosphate, a chemical substance that, when present on the surface of implants, provides a conductive microenvironment, contributing to bone growth.^1^

A characteristic of titanium is that when exposed to body fluids, it stimulates the growth of hydroxyapatite, which is essential for promoting implant osseointegration.^2–5^

Blood and other components of body fluids such as water, proteins, chlorine and sodium make the human body a highly corrosive environment, which is undesirable for materials as it affects mechanical resistance and facilitates the release of toxic residues into the body. Each metal has a different reactivity, and some metals develop an oxide layer on their surface when exposed to extreme environments or air. Titanium is very reactive and has an oxide layer that adheres strongly to the surface of implants, reducing the transfer of ions from the metal to body fluids and protecting against corrosion.^6^

However, the use of metals without surface modification is not recommended, since the oxides they form when in contact with air are unstable. The resulting thin oxide layer has high porosity and low mechanical resistance, and thus does not protect the metal from corrosion.^7^

Therefore, modification of the titanium surface of implants is necessary to acquire good properties, which is of utmost importance as it increases the surface energy responsible for surface roughness and the desired chemical composition. This also increases osseointegration and adhesion to tissue, in addition to reducing bacterial reactions and inflammatory responses in the body. To achieve this surface modification, the anodizing method is commonly used.^8^

In the production of highly ordered TiO_2_ nanotubes (TNT) by anodizing, the electrolytes used are composed of fluorinated substances such as HF, NaF, NH_4_F and an aqueous support solution containing H_3_PO_4_, H_2_SO_4_, glycerol, NaHCO_3_ or Na_2_SO_4_. The two types of components combined are of vital importance, since the use of the support electrolyte without fluorine does not form TNT, only a compact TiO_2_ oxide layer.^8^

On the other hand, when using the fluorinated compound alone, the oxidation and hydrolysis reactions of titanium do not occur uniformly, i.e. a disordered nanoporous structure is obtained.^9,10^

In contrast, concerns about environmental issues and the health of process operators who are exposed to fluoride and potentially polluting chemical effluents are a deadlock. Industries often have difficulty meeting the effluent discharge standards required by law. If excessive amounts are released into the environment, they cause eutrophication (in the presence of compounds such as nitrogen and phosphorus) and increased toxicity in water resources, affecting the health of aquatic species and humans.^11^

However, the use of electrolytes that are less harmful to the environment and human health, such as plant extracts, has gained prominence due to their environmentally friendly processes, in addition to the ample availability of natural resources and their low cost.^12^ In this sense, plant extracts are rich sources of organic compounds, and in the case of *Psidium guajava* leaves, their two main compounds, quercetin and ascorbic acid, have in their structures a large number of oxygen atoms in functional groups and aromatic rings, which are important for adsorption to the metal surface and consequently the formation of an anticorrosive biofilm.^13^ The oxidizing effect during anodizing is mainly due to the presence of phenolic compounds based on C, H and O in *Psidium guajava* leaves. Through the combination of −OH species from the electrolyte that come into contact with the metal, titanium oxide is formed.^12^

Seeking to mitigate harmful effects on the environment and the health of process operators, the central objective of this study is to compare the anodizing of CP2 titanium using a more sustainable, *Psidium guajava*-based organic electrolyte with the industrial anodizing process in two configurations, H_3_PO_4_ 1 M and H_3_PO_4_ 1 M + 0.15% v/v HF.

## 2 Experimental Section

### 2.1 Preparation of Electrolytes

For the electrolyte containing only phosphoric acid (H_3_PO_4_), the concentration used was 1 M and for phosphoric acid + hydrofluoric acid (H_3_PO_4_ + HF), the concentration was H_3_PO_4_ 1 M + HF 0.15% w/v. Regarding the *Psidium guajava* electrolyte, the leaves were leaf collection was performed manually in the *Psidium guajava* orchard, located on Feevale University’s Campus II, coordinates 29°40′00’’S and 51°07′10’’W, and were washed and dried in an oven with air circulation for approximately 3 days at a temperature of 45 °C ± 5 °C. After drying, the leaves were ground until powder was obtained. A J-BM1-S ball mill was used to grind the leaves at a speed between 68 and 80 rpm. After this period, the ground leaves were placed on a 400-mesh sieve to achieve uniform particle size distribution. To prepare the plant extract, 6 g of the powder were weighed and 120 mL of ultrapurified water were added. Subsequently, the solution was heated to 40 °C ± 2 °C with stirring (n° 3), remaining for 15 min at the appropriate temperature. After cooling, filtration was carried out, obtaining 90 mL of electrolyte with a pH of 5.

### 2.2 Anodizing

The anodizing processes were performed with three different electrolytes, H_3_PO_4_, H_3_PO_4_ + HF and *Psidium guajava*, using commercially pure titanium sheets, grade 2 (ASTM B348), pickled in dimensions of 2.5 cm × 7 cm with 1 mm thickness through the potentiostatic method, in a potential x current source (0–300 V, 0–500 mA). The samples were pickled with acids to remove impurities and prepare them for anodizing. All samples were immersed in a 40% HF (hydrofluoric acid) + HNO3 (nitric acid) solution for 1 min, under agitation, to clean them. Since the aim was to repeat the industrial process, the parameters of 15 V for 10 s were used for the anodizing processes in H_3_PO_4_ and H_3_PO_4_ + HF. For the *Psidium guajava* samples, the same potential of 15 V was used, but with a time of 15 s, in order to obtain a similar coloration on the anodized surface. All anodizing processes were performed without agitation of the electrolytes. The cell volume used was 100 mL. The anodizing process was conducted in a 100 mL beaker, with titanium used as the working electrode and, in parallel, two counter electrodes, also on both sides, spaced 1 cm apart. The pH was measured before anodizing, at pH = 5, and after anodizing, dropping to pH = 4. The temperature used was room temperature. The potential increased linearly, remaining constant at 15 V throughout the process, and the current density also increased at the beginning of the process and then decreased rapidly, remaining low throughout the process.

### 2.3 Characterizations

Morphological analyses were performed in top view to evaluate the coverage of the anodized layer and combined with corrosion tests to evaluate the surface after immersion in SBF (simulated body fluid). The SEM (scanning electron microscopy) equipment used was the JSM-6510LV model, Jeol brand. As a process parameter, 20 kV with 1000× magnification was used for all samples, and in the case of the H_3_PO_4_ + HF sample, 5000× magnification was added. After the electrochemical tests, the samples were analyzed to assess changes in morphology via SEM, using an acceleration voltage of 10 kV, with magnifications of 1000x for comparative purposes and 3000x. The roughness tests were performed in triplicate using a mechanical profilometer, Ambios brand, model XP-2, with a sample size of 1 cm^2^. The following parameters were used: Ra (arithmetic mean of the absolute deviations of the roughness profile in relation to the mean line within the sampling length), Rq (square root of the mean of the squares of the deviations of the roughness profile in relation to the mean line within the sampling length), Rt (total roughness height, defined as the vertical distance between the highest peak and the deepest valley within the sampling length), Rz (sum of the means of the five highest peak heights and the five highest valley depths within the sampling length), RyJIS (parameter defined by the Japanese standard JIS B0601, which measures the maximum height of the profile) and Rmax (maximum roughness depth measured as the vertical distance between the highest peak and the deepest valley within a single evaluation length). The samples anodized in H_3_PO_4_, H_3_PO_4_ + HF and *Psidium guajava* were evaluated for their visual coloration and compared with the literature considering the process parameters and the colors of the oxides formed. The electrochemical tests were performed using the PGSTAT302 potentiostat by Autolab. To this end, a three-electrode cell containing a working electrode (unanodized and anodized titanium sheets), a reference electrode (SEC – saturated calomel electrode) and a counter electrode (platinum grid) was used. The SBF electrolyte was used to simulate the corrosive behavior to which the material is exposed in the human body, at a pH of 7.4 and room temperature, following the proportions described by Fernandes et al. (2022).^13^ The open circuit potential (OCP) was monitored at 24, 48, 72, and 96 h of immersion. The OCP values were obtained before the electrochemical impedance spectroscopy (EIS) measurements, from the same electrolyte. The EIS measurements were performed using the OCP with a 10 mV sinusoidal signal and a sweep from 100 kHz to 10 mHz. The analyses were performed with immersion time intervals in SBF of 24, 48, 72, and 96 h. The data analysis was performed using the software Origin, with the preparation of the Bode plot divided into two diagrams to show the behavior of the phase and the impedance module separately. The μ-Raman spectroscopy tests were performed using the NanoLabRAM Confocal Raman microscope by HORIBA. Since the titanium samples anodized in *Psidium guajava* showed satisfactory electrochemical behavior for corrosion resistance, which was suggested by the presence of the electrolyte compounds, the μ-Raman analyses were carried out only for the samples of as-received pure titanium (without anodizing) and *Psidium guajava*-anodized titanium. The process parameters used were laser excitation at 532 nm and scanning up to 3000 cm^–1^. The characterization of phenolic compounds was identified through Raman spectrophotometry, NanoLabRAM Confocal Raman microscope by HORIBA, which accurately identifies the presence of specific compounds, in this case phenolics, and is widely used for this quantification. The photocatalytic performance of the anodized semiconductors was observed based on the discoloration of the methylene blue dye concentration under UVC irradiation. Tests were performed in triplicate. The analyses were performed in a Pyrex glass photocatalytic reactor, where radiation was provided by a 9 W Philips black UV–C lamp. The radiation intensity during the tests was 1.34 W/cm^2^. The lamp was arranged to ensure uniform illumination of the semiconductors. The other components of the photocatalytic reactor include a magnetic stirrer and a thermostatic bath. First, a solution containing 20 ppm of methylene blue dye and 100 mL of deionized water was prepared. Before beginning the photocatalytic analyses, adsorption/desorption tests were performed, where the mixture was placed and kept in a dark place for 15 min to demonstrate that no particles adsorbed on the semiconductor surface. The resulting solution was then transferred to the reactor flask, where the semiconductors were supported one at a time. Before each test, a 4 mL sample of this solution was collected as the initial sample. During the test, with the UV light system on, 4 mL aliquots of the solution were collected at 30 min intervals and placed in volumetric flasks for analysis using a Femto Cirrus 80 ST spectrophotometer. Before beginning the spectrophotometric analysis, the aliquots were transferred from the volumetric flask to quartz cuvettes. The band gap energy was determined by the Kubelka and Munk correlation associated with the formula E­(eV) = 1240/λ (nm), using diffuse reflectance spectroscopy (DRS) data obtained using a Thermo Scientific UV–vis spectrophotometer, model Evolution Pro, with the diffuse reflectance accessory. The above formula is derived from the relationship between the energy and frequency of a photon, considering Planck’s constant and the speed of light, where

1. E is the energy in electron volts (eV) and
2. λ is the wavelength in nanometers (nm).

## 3 Results and Discussion

### 3.1 SEM Morphological Analysis

The micrographs in Figure 1 show the morphology of the as-received and pickled titanium samples in top view.

**Figure 1:**
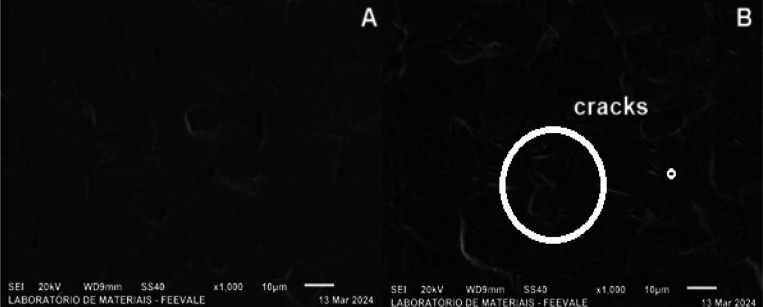
Top-view micrographs of the (a) as-received and (b) pickled titanium samples. Source. Authors, (2024).

Figure 1 shows the titanium grains in the as-received sample (a), which become more defined after pickling (b), which also generates cracks on the surface. According to Kurup et al., 2020,^14^ the pickling process removes oxides and dirt from the metal surface. We obtained similar results to the study by Contieri et al., 2010.^15^

According to the authors, the micrograph indicates that the titanium sample was annealed, as it shows grains of approximately 10 μm. This is in agreement with the micrographs obtained in this research, which suggests that the titanium used in the present study was also annealed.

However, after the pickling process, cracks are observed on the surface of the sample. According to Sutter and Goetz-Grandmont (1990),^16^ Ti has excellent corrosion resistance in many dilute acidic media, except in hydrofluoric acid, since it dissolves the metal according to eq 1.

$$\mathrm{Ti}+3\mathrm{HF}\to {\mathrm{Ti}}^{3+}+3/2H_{2}+3F^{-}$$ 1

Ti^3+^ can be subsequently oxidized by atmospheric oxygen (or, very slowly, by HF itself) and complexed by F-. However, the use of pure hydrofluoric acid as a pickling solution for titanium forms hydride on the surface of the metal, which promotes embrittlement. Likewise, the application of pure nitric acid causes the passivation of titanium, with the formation of a passive protective layer of TiO_2_ on its surface. For this reason, industrial pickling of Ti is commonly done in an aqueous solution of nitric and hydrofluoric acid (Sutter et al., 1990).^16^

Thus, it is observed that HF embrittles the titanium surface due to the formation of hydride and consequently the release of hydrogen, which could cause a decrease in local pH and the formation of the cracks observed in Figure 1(b).

On the other hand, HNO_3_ causes titanium passivation, suggesting the use of a nitric-hydrofluoric acid solution, which is consistent with the solutions used in the experiments to clean the surface before anodizing.

The micrographs in Figure 2 show the morphology of titanium anodized in H_3_PO_4_ and in *Psidium guajava* in top view.

**Figure 2:**
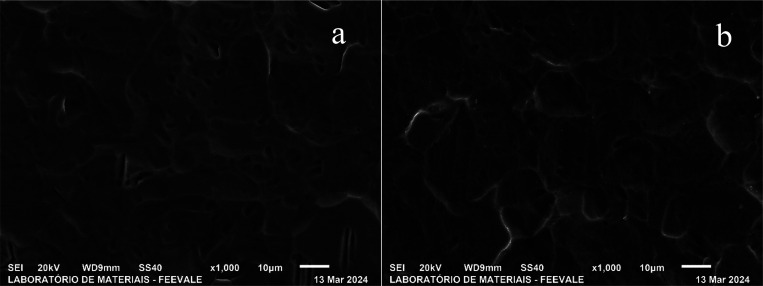
Top-view micrographs of titanium anodized in (a) H_3_PO_4_ and (b) *Psidium guajava*.

In Figure 2, a similar morphology can be observed for titanium anodized in both H_3_PO_4_ (a) and *Psidium guajava* (b). Furthermore, although both samples have cracks similar to those of the pickled sample (Figure 1b), it can be seen that the cracks became convex at the anodized ends.

In the study by Yilmaz et al., (2023),^17^ photocatalytic TiO_2_ coatings were obtained via anodizing with an electrolyte composed of 28% HNO_3_, 1% HF and 71% H_2_O, in addition to a voltage of 20 V with different process times. The imperfections on the titanium surface were not significantly changed after 10 s of anodizing, only after 30 s. Therefore, the authors concluded that the changes in surface morphologies depended on the process time and that 10 s of anodizing were not enough to provide a new surface morphology, indicating that the oxide film is quite thin. These results are in agreement with the micrographs in Figure 3, which shows that the oxide formed in both H_3_PO_4_ and *Psidium guajava* is tenuous. On the other hand, it is worth noting that although the TiO_2_ film is quite thin, there are stresses generated in the oxide during its formation, which could explain the more “rounded” appearance of the cracks.

**Figure 3:**
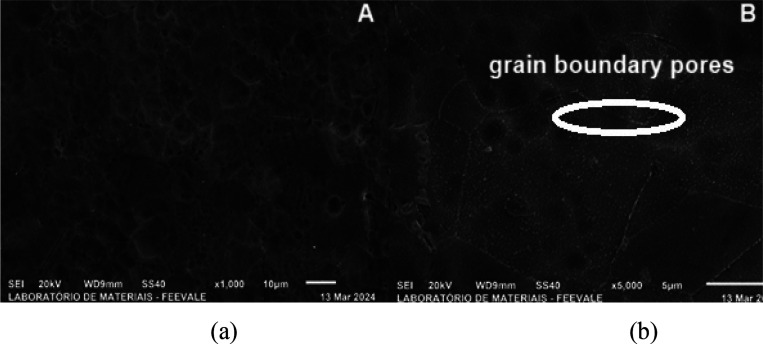
Top-view micrographs of titanium anodized in H_3_PO_4_ + HF.

During anodic oxidation, compressive deflection occurs due to electrostriction, which increases linearly with the potential during oxide growth. Anodic oxides are dielectrics, and the electric field exerts a force perpendicular to the plane of the oxide layer. As the oxide is compressed, it tends to expand in the plane of the film, but is restricted by the metal and thus develops lateral stress. Therefore, oxides that grow slowly show greater electrostrictive deflection than fast-growing oxides. Additionally, one must also consider the tensile stress that develops at the metal/oxide interface due to the difference in volume between the ionized metal and the oxide formed at this interface (Nelson et al., 1993).^18^ In the case of the present study, the formation of the oxide generates lateral stress, which would be responsible for the “rounding” of the cracks. Figure 3shows the morphology of titanium anodized in H_3_PO_4_ + HF in top view, with magnifications of 1000x (a) and 5000x (b).

Figure 3 shows that the micrograph of titanium anodized in H_3_PO_4_ + HF is different from that of Figure 3(a), which refers to titanium anodized only in H_3_PO_4_. Small porosities created by the addition of HF are observed mainly at 5000× magnification (Figure 3b).

According to Roy et al., (2011),^19^ during the formation of titanium oxide, species migrate from the electrolyte to form the oxide. In the case of fluoride, [TiF_6_]­2- is formed, which is soluble in water and thus explains the generation of pores. Furthermore, in the study carried out by Kunst et al., (2020),^20^ porosity was formed in titanium anodized in 1 M H_3_PO_4_ + 0.15% HF and the pore diameter ranged from 61 to 76 nm ± 10 nm. Even so, the thickness of the oxide was quite thin as it was not enough to cover the titanium grain, since the authors identified pores in the grain boundary, in agreement with the morphology obtained in the present study, as shown in Figure 3(b). Although the oxide formed is thin, it appears to be thicker than those anodized without HF, since it does not faithfully “copy” the surface, which is the characteristic behavior of the formation of porous oxide.

### 3.2 Roughness Analysis

After the morphology analyses, a roughness test was performed to complement the surface results. According to Pavei et al., (2022),^21^ a rough surface is very important for cell growth and increased bone formation in the implant area. The mean and standard deviation of the results for the roughness parameter are presented in Table 1.

**Table 1:** Roughness Measurements of As-Received, Pickled, and Anodized Titanium Samples in H_3_PO_4_, *Psidium guajava*, and H_3_PO_4_ + HF

sample	Ra (μm)^a^	Rq (μm)^b^	Rt (μm)^c^	Rz (μm)^d^	RyJIS (μm)^e^	Rmax (μm)^f^
received	0.120 ± 0.014	0.163 ± 0.019	1.264 ± 0.224	0.471 ± 0.042	1.264 ± 0.224	1.234 ± 0.222
pickled	0.271 ± 0.009	0.353 ± 0.011	2.569 ± 0.183	0.960 ± 0.018	2.569 ± 0.183	2.277 ± 0.128
H_3_PO_4_	0.247 ± 0.031	0.318 ± 0.031	2.153 ± 0.145	0.903 ± 0.083	2.153 ± 0.145	1.973 ± 0.352
*Psidium guajava*	0.247 ± 0.009	0.317 ± 0.012	2.334 ± 0.192	0.873 ± 0.044	2.334 ± 0.192	2.100 ± 0.025
H_3_PO_4_ + HF	0.100 ± 0.010	0.125 ± 0.012	0.875 ± 0.170	0.395 ± 0.023	0.875 ± 0.170	0.820 ± 0.208

a Ra: the arithmetic mean of the absolute deviations of the roughness profile from the mean line within the sampling length.

b Rq: the square root of the mean of the squares of the deviations of the roughness profile from the mean line within the sampling length.

c Rt: the total roughness height, defined as the vertical distance between the highest peak and the deepest valley within the sampling length.

d Rz: the sum of the means of the five highest peak heights and the five highest valley depths within the sampling length.

e RyJIS: a parameter defined by the Japanese standard JIS B0601, which measures the maximum height of the profile.

f Rmax: the maximum depth of roughness measured as the vertical distance between the highest peak and the deepest valley within a single evaluation length.

Evaluating the results described in Table 1, the as-received titanium has a surface roughness of Ra = 0.120 ± 0.014 μm, similar to the value found by SafaviPour et al. (2023),^22^ which was 0.132 ± 0.026 μm for nonanodized titanium.

For the pickled titanium sample, there was an increase in all roughness parameters, practically double the value, compared to the as-received titanium. Through the pretreatment and anodizing processes, it is possible to change the roughness, microstructure and porosity of a material in order to improve the metal-tissue integration. The study by Shabani (2015)^23^ proved that the use of a chemical attack process with a pickling solution of nitric and hydrofluoric acid promoted a greater average surface roughness before and after anodizing when compared to other methods such as polishing. The chemical attack of the titanium surface using acidic solutions such as nitric and hydrofluoric acid produces microcavities and thus alters the surface morphology, which justifies the higher roughness values observed.^24^ This result is in agreement with the formation of cracks and passivation observed by SEM (Figure 4b).

**Figure 4:**
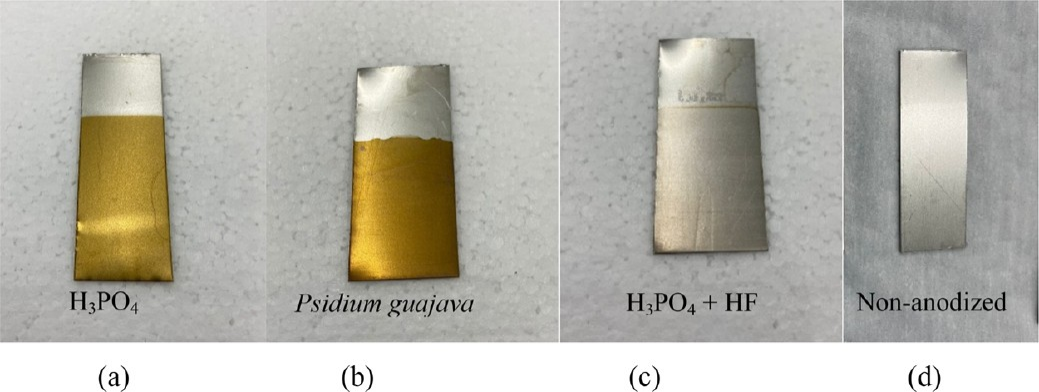
Coloration of titanium anodized in (a) H_3_PO_4_, (b) *Psidium guajava*, (c) H_3_PO_4_ + HF, and (d) without anodizing.

According to Jahani and Wang (2021),^25^ the range of surface roughness (Ra) values found by different authors is wide, showing results from 0.07 to 100 μm. This discrepancy occurs because roughness can play a dual role in orthopedic implants, i.e. it can improve osseointegration and, at the same time, increase the loss of mechanical properties.

Another consideration to be made is microbial activity and roughness. According to Reidel et al., (2025),^26^ roughness influences the accumulation of bacterial plaque, with a threshold of 0.2 μm for the recommended roughness for the surface of the biomaterial, which would be in agreement with the samples anodized in H_3_PO_4_ and *Psidium guajava*. Moreover, both samples form very fine oxides with little variation in surface roughness (compared to the pickled sample), since they practically “copy” the surface of the pickled titanium, in agreement with the micrographs obtained by SEM (Figure 3). In the case of H_3_PO_4_ + HF, a metal complex with fluorine is formed that is soluble in solution and is responsible for the porosity of the oxide. However, this porosity is not detected by the roughness technique. In this case, since the roughness results obtained were lower among the anodized samples (Ra = 0.100 ± 0.010), it is assumed that the oxide fills the surface imperfections due to the formation of a porous oxide. This is in agreement with the micrographs obtained by SEM in which porosities were observed and no cracks were seen, as shown in Figure 4.

### 3.3 Visual Chromatic Analysis

Saraswati et al. (2020)^27^ obtained different colors for Ti-6Al-4 V titanium alloy samples anodized at different potentials (10 V, 20 and 30 V) in a KOH electrolyte for 20 s. The colors obtained were gold, dark blue and light blue, respectively. In addition, research by Fuhr et al., (2024)^28^ also assessed the coloration resulting from the galvanostatic anodizing of grade 2 titanium in a pyroligneous liquor electrolyte, with a current density of 1 mA.cm^–2^, at anodizing times of 300, 600, 1800, and 3600 s.

The pyroligneous liquor has a reddish-brown coloration and potassium hydroxide is a colorless electrolyte, i.e. the coloration shown by the samples is not linked to the coloration of the electrolyte but rather to the one acquired during anodizing. The phenomenon of light interference is responsible for the particular coloration of the anodized samples, and it is related to the increase in thickness of the oxide formed and the variation in potential.^28,29^ The *Psidium guajava* electrolyte is a reddish-brown and translucent electrolyte, while the H_3_PO_4_ and H_3_PO_4_ + HF electrolytes are colorless.

The coloration of the anodizing obtained by (a) Saraswati et al., (2020)^27^ and (b) Fuhr et al., (2024)^28^ are in line with those obtained from the experimental procedure on titanium anodized in H_3_PO_4_ and in *Psidium guajava*, as illustrated in Figure 4.

Regarding the H_3_PO_4_ + HF electrolyte, the coloration of anodizing was more subtle, presenting a lighter yellowish coloration when compared to the titanium sample without anodizing (only pickled). This may be related to the anodizing time and/or the dissolution of the oxide layer caused by the fluoride.^30^ Petry et al., (2024)^31^ also verified the creation of yellow oxides formed in titanium under anodizing conditions of 600 s at a current density of 2 mA/cm^2^ and free voltage, with the electrolyte being agitated at 100 rpm; this formation was attributed to the low oxide thickness of the sample. It can be concluded that the coloration observed is related to the applied potential, regardless of the electrolyte, agreeing with the coloration results obtained in the literature for low potential.

### 3.4 Electrochemical Corrosion Tests

#### 3.4.1 Electrochemical Impedance Spectroscopy (EIS)

The plots in Figure 5 demonstrate the behavior of titanium during the impedance test for the electrochemical corrosion test in SBF. The left plot represents the values for log |Z| x log f, and the right plot represents the phase graphs (phase x log f).

**Figure 5:**
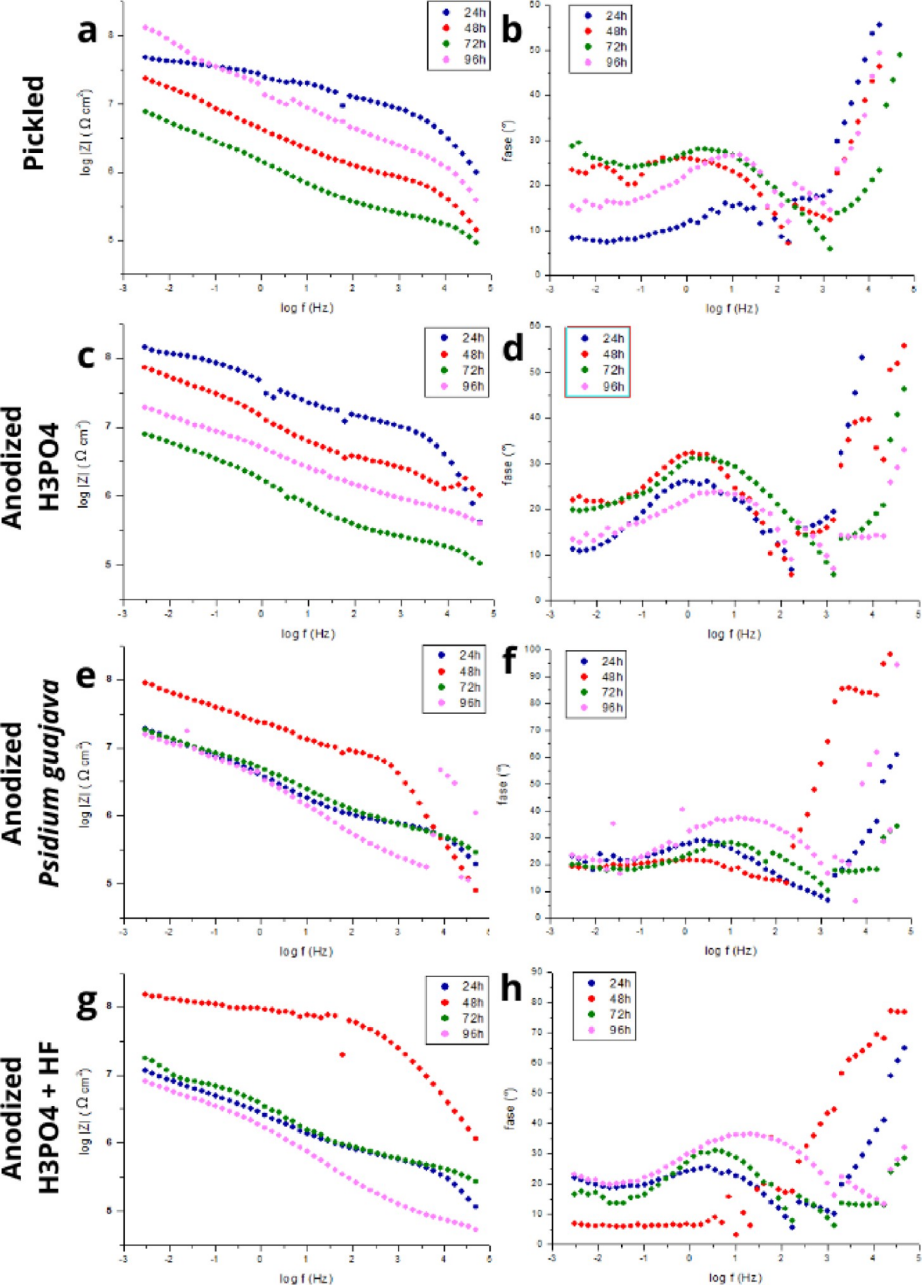
Bode plots for pickled and anodized samples immersed in SBF for 24, 48, 72, and 96 h. (a, b) Pickled, (c, d) anodized H_3_PO_4_, (e, f) anodized *Psidium guajava*, and (g, h) anodized H_3_PO_4_ + HF.

The impedance of the SBF-immersed samples was not measured at the 1 h mark due to the instability of the measurements, following Kunst et al., (2021),^32^ who obtained these instabilities for anodized titanium. Therefore, measurements were performed at 24, 48, 72, and 96 h to evaluate impedance.

In Figure 5 (a,b), phase graph (b), a high-frequency phenomenon associated with the passivation of the metal during the pickling process is observed at all immersion times, which was evidenced in the micrographs obtained by SEM. The formation of the passive layer using nitric acid results in the passivation of the titanium, with the formation of a passive protective layer of TiO_2_ on the metal surface. However, it can be noted that at all immersion times, there also occurs a medium- to low-frequency phenomenon with a low phase angle associated with the permeation of the electrolyte through the cracks in the passive layer. This was possibly due to cracks obtained during the pickling process, as well as the formation of hydride and consequent embrittlement of the surface previously mentioned in the SEM test in Figure 1 (b), which possibly contributed to facilitating the entry of ions and accelerating the corrosion process.

It is observed that diffusion occurs at a phase angle of 30°; under these conditions, the diffusion component is typical of a surface containing corrosion products that act as a barrier. This occurs at the passive film/solution interface, where there is less chance of solution entry through corrosion products.^32^ This is probably due to the instability of TiO_2_, which promotes corrosion products that block the active sites on the surface. The stability of the oxide in the human body is also temporary, as evidenced by Paim et al., (2023),^33^ who observed that the oxide begins to dissolve over time when immersed in SBF.

The use of SBF is the most common form of hydroxyapatite (HAp) deposition. At a pH of 7.4, titanium–OH groups are negatively charged due to the deprotonated acidic hydroxides in the medium. Calcium ions are adsorbed to the SBF on the metal surface, and calcium reacts with H_2_PO^4–^ to form calcium phosphate. Furthermore, according to Versteg et al., (2019),^34^ this HAp layer dissolves in an acidic medium, following eq 2.

$${\mathrm{Ca}}_{10}({\mathrm{PO}}_{4}{)}_{6}{\mathrm{OH}}_{2}+2H^{+}\to 10{{\mathrm{Ca}}_{2}}^{+}+6{{\mathrm{PO}}_{4}}^{3-}+H_{2}O$$ 2

The author also observed flaws in the passive layer that became preferential paths for the electrolyte on the titanium metal surface, causing corrosion^34^ and confirming the results obtained in the present study (with diffusion observed at a phase angle of 30°), since corrosion products are produced and solubilized as the time of immersion in SBF increases.

According to Alves et al., (2009),^35^ another reaction of corrosion products in SBF is the formation of soluble titanium chlorocomplexes. Passivating biomaterials such as titanium can generate metal ions that react with body fluids, especially with chloride. The formation of a soluble titanium chlorocomplex is also supported by the passivation mechanism, which is the result of the hydrolysis of titanium chloride in aqueous solutions forming a passive TiO_2_ film, as described in eq 3.

$$\mathrm{Ti}\to {\left[{\mathrm{Ti}\mathrm{Cl}}_{6}\right]}^{2-}↔{\mathrm{TiO}}_{2}+6{\mathrm{Cl}}^{-}+4H^{+}$$ 3

Some of the corrosion products and ion fluxes formed in SBF were summarized in Figure 6.

**Figure 6:**
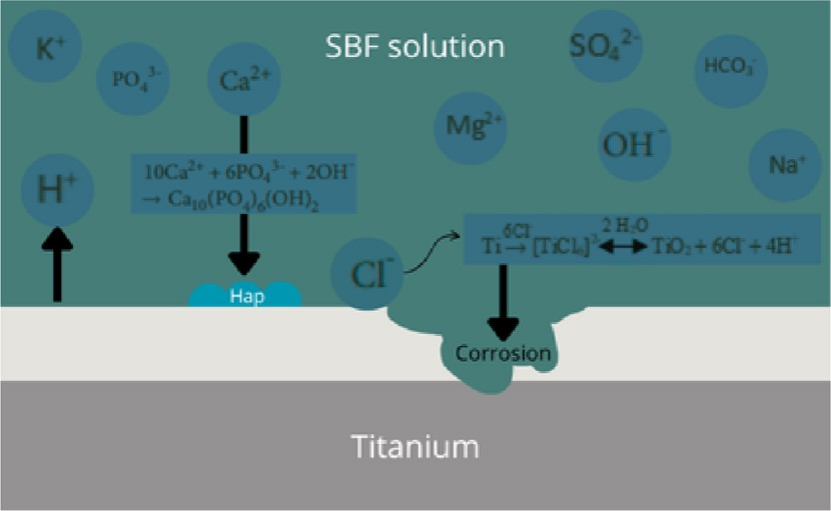
Corrosion products and ions in SBF.

Figure 5 (c,d) shows two phenomena, one at high frequency and the other at medium frequency, with a low phase angle. Upon closer analysis, it can be seen that in the sample anodized in H_3_PO_4_, unlike in the results for the pickled sample (Figure 5 a,b), the phenomenon at low frequency associated with corrosion products in a defined form is not observed, since they maintain the same phase angle at medium and low frequency. According to Liu et al., (2010),^36^ anodized titanium is expected to have a phase angle close to 90°, with capacitive behavior. Titanium oxide generally shows a time constant from medium to low frequency associated with the capacitive performance of the oxide, which was not observed in the sample anodized in H_3_PO_4_. The low anticorrosive performance of the anodized layer (Figure 5 c,d) may be due to the extremely thin oxide formed, since the anodizing procedures were performed at a low potential of 15 V for 10 s and did not demonstrate a protective character. The type of electrolyte and pH, anodizing time and applied potential affect the crystallinity and morphology of the surface,^37^ as verified in the results shown in Figure 5 (c,d). In the titanium anodized in *Psidium guajava*, Figure 5 (e,f), two phenomena are also observed, one at high and one at medium frequency. At 24 h of immersion, the medium-frequency phenomenon shows similar behavior to that of the sample anodized in H_3_PO_4_ with a phase angle close to 30° and frequency around 100.5 Hz. As the time increases to 48 h, this medium-frequency phenomenon decreases its phase angle to approximately 20° and the high-frequency phenomenon becomes more evident. This indicates the formation of corrosion products on the metal surface. At 72 h, the high-frequency phenomenon increases the phase angle again to 30° and shifts to a frequency of 10 Hz. At 96 h, an increase in the phase angle to 40° at medium frequency is observed, with its shift to 101.5 Hz and the emergence of the high-frequency phenomenon again. This behavior is associated with the corrosion products that temporarily protect the electrolyte coating. These results agree with the study by Silva et al., (2024),^38^ which found an increase in the resistance of the corrosive product layer proportional to the time of immersion in the electrolyte, justifying that the corrosion products act as a barrier to the permeation of the electrolyte. Regarding the titanium sample anodized in H_3_PO_4_ + HF, Figure 5 (g,h) shows two phenomena after 24 h of immersion, one at medium frequency and the other at low frequency, similar to the samples anodized in H_3_PO_4_ (Figure 7 c,d) and *Psidium guajava* (Figure 5 e,f). As the immersion time increases to 48 h, the medium-frequency phenomenon almost disappears and the high-frequency phenomenon becomes more evident. At 72 h of immersion, the medium-frequency phenomenon increases, returning to the behavior shown at 24 h, and the high-frequency phenomenon decreases. At 96 h, the medium-frequency phenomenon increases and shifts the phase angle to higher frequencies, similar to the behavior observed in *Psidium guajava.*

**Figure 7:**
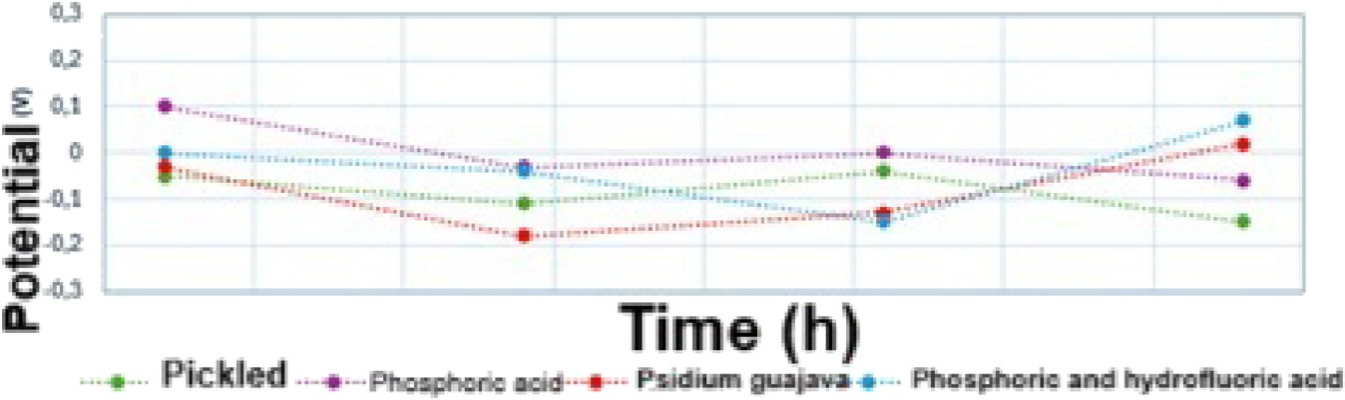
Open circuit potential.

Comparing the samples anodized in H_3_PO_4_ + HF with those anodized in *Psidium guajava*, it can be stated that the *Psidium guajava* samples had better electrochemical performance. This may be associated with the oxidizing effect of *Psidium guajava* during anodizing, which occurs mainly due to its phenolic compounds.

Polyphenolic compounds are secondary metabolites of plants and have hydroxyl groups and aromatic rings, with a varied chemical structure. In a study by Clemes et al., (2015),^39^ the aqueous and ethanolic extracts of *Psidium guajava* showed little difference in relation to the number of polyphenols extracted and the antioxidant capacity of both extracts. Therefore, these compounds can be obtained using an aqueous electrolyte, which agrees with Silva et al. (2024)^38^ since aqueous extracts of guava leaves are rich in antioxidant phenolic compounds. Among phenolic compounds, flavonoids have a chemical structure with two aromatic rings, which confer antioxidant characteristics. This occurs due to reactivity, which is capable of sequestering free radicals and metals with chelation (covalent bond between a metal and organic compounds), as well as preventing or reversing oxidative processes.^40^

The occurrence of metal chelation is linked to the arrangement of hydroxyl and carbonyl groups around the molecule. The reduction of free radicals is driven by the presence of electron or hydrogen donating substituents, and the formation of phenoxyl radicals depends on the displacement of the unpaired electron from the molecule, making them less available in the reaction medium.^41^ Therefore, the phenolic compounds present in the *Psidium guajava* plant extract interact with the metallic surface of titanium and, when a potential or current is applied, act as oxidants during anodizing, being consumed in this process, which justifies their better electrochemical performance.^41^

#### 3.4.2 Open Circuit Potential (OCP)

The open circuit potential (OCP) was measured at 24, 48, 72, and 96 h of immersion in SBF, prior to the EIS tests, for the pickled (without anodizing) titanium sample, as well as those anodized in phosphoric acid (H_3_PO_4_), phosphoric acid + hydrofluoric acid (H_3_PO_4_ + HF) and *Psidium guajava*, resulting in the measurements shown in Figure 7.

For the pickled sample (Figure 7 - 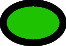), a small oscillation of the OCP value was observed alongside immersion time. This small oscillation occurred for more negative OCP values (at 48 and 96 h), indicating the formation of passivating corrosion products. This is due to the formation of a protective oxide layer, such as TiO_2_ or corrosion products, which reduce the corrosion rate by acting as a barrier between the metal and the SBF solution, leading to a more stable potential over time.^42^ This behavior is in accordance with the EIS results in Figure 5 (a,b), since corrosion products are formed and solubilized as the time of immersion in SBF increases.

Regarding the titanium sample anodized in H_3_PO_4_ (Figure 7- 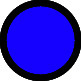), its potential is at first positive during the initial 24 h of immersion, then it decreases in the next measurement, at 48 h, acquiring negative results. At 72 h, the potential value was 0 V, and at 96 h it was negative again, reaching −0.06 V. This is in agreement with the oxide formed being very thin, as per the EIS results in Figure 5 (c,d). According to Kunst et al., (2021),^32^ high OCP values are temporary due to the instability of the oxide in the human body, which is explained by its dissolution with increasing time of immersion in SBF, suggesting a broad interaction between the anodized coating and the body fluid. The authors concluded that anodized samples, compared to pure titanium, have decreased susceptibility to corrosion and increased bioactivity.

Regarding the titanium samples anodized in *Psidium guajava* (Figure 7 - 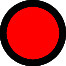) and in H_3_PO_4_ + HF (Figure 9 - 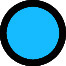), a decrease in the OCP value is observed up to 48 h for *Psidium guajava* samples and up to 72 h for H_3_PO_4_ + HF samples, followed by an increase in the OCP value, with both electrolytes having a positive potential after 96 h of immersion. This behavior is explained by SafaviPour et al., (2023),^22^ who also observed similar behavior in the OCP analysis for titanium. According to the authors, this phenomenon occurs due to the penetration of the solution into the coating layer, followed by the creation of corrosion products at the coating/substrate interface act as a barrier to electrolyte permeation. Likewise, the *Psidium guajava* samples showed better anticorrosive performance (in agreement with the EIS results in Figure 5 (e,f)) due to the oxidizing effect of *Psidium guajava* in anodizing, which is related mainly to the effect of its phenolic compounds.

### 3.5 SEM Morphology after Exposure to SBF

After the EIS tests, morphological analyses were carried out using SEM to compare them with samples that were not exposed to SBF. For comparison purposes, the morphologies of the samples that were not exposed to SBF (discussed in item 3.1) will be repeated together with the micrographs taken after exposure in SBF. shows the morphology of the pickled titanium a) without exposure to SBF (Figure 1b) and b) after 96 h of exposure to SBF.

Produtos de corrosão

According to Figure 8, the formation of corrosion products is observed after exposure to SBF. In addition, the cracks originated from the pickling process (Figure 8a) are smoothed after exposure to SBF, indicating that corrosion products are formed due to the permeation of the electrolyte into the surface cracks, in agreement with the results of Figure 5 (a,b) obtained in the impedance and OCP tests (Figure 7 - 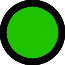).

**Figure 8:**
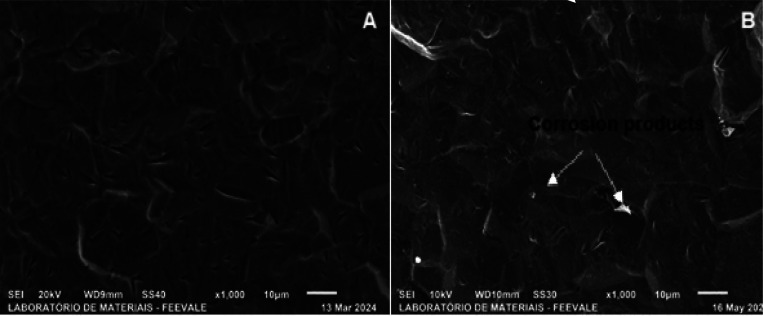
Top-view micrographs of pickled titanium (a) before (Figure 1b) and (b) after 96 h of immersion in SBF.

Figure 9 shows the sample anodized in H_3_PO_4_ (a) without exposure to SBF (Figure 3a) and (b) after 96 h of exposure to SBF.

**Figure 9:**
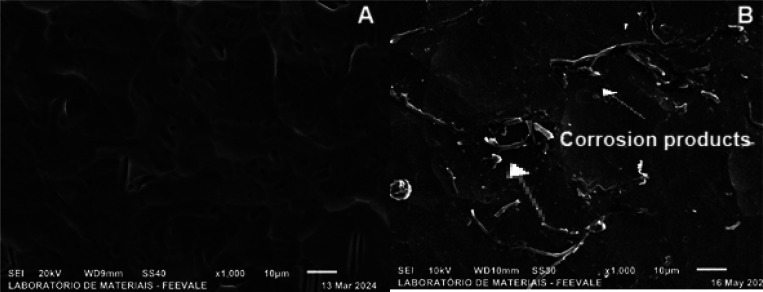
Top-view micrographs of titanium anodized in H_3_PO_4_ (a) before (Figure 1a) and (b) after 96 h of immersion in SBF.

It can be seen in Figure 9 (a) that the cracks from the pickling process, which were rounded due to anodizing, were completely covered by the corrosion products (Figure 9 b); also visible are heterogeneous depositions on the sample surface. This result agrees with those of the electrochemical impedance (Figure 5 c,d) and OCP tests (Figure 9 - 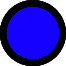), in which interaction between the anodized coating and the body fluid was observed, with the formation of corrosion products.

Figure 10 shows the sample anodized in *Psidium guajava* (a) without exposure to SBF with a magnification of 1000x (Figure 1b), and (b) (c) after 96 h of exposure to SBF with magnifications of 1000x and 3000x, respectively.

**Figure 10:**
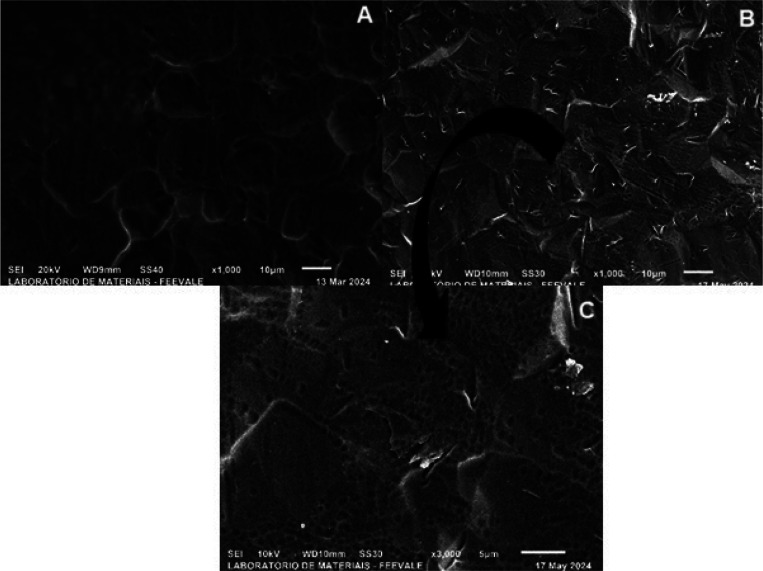
Top-view micrographs of titanium anodized in *Psidium guajava* (a) before immersion in SBF at 1000× magnification (Figure 3b), and (b, c) after 96 h of immersion in SBF at 1000× and 3000× magnification, respectively.

b

In Figure 10, it can be observed that the sample anodized in *Psidium guajava* (a) before and (b) after 96 h of exposure to SBF showed little difference in morphology at 1000× magnification. The presence of corrosion products was verified, however; unlike the H_3_PO_4_ sample, cracks were still seen after exposure to SBF and the pores became more defined, as observed in Figure 10 (c). This characteristic of *Psidium guajava* can be attributed to the phenolic compounds of the electrolyte, which aid in its anticorrosive performance, as already described in the EIS (Figure 5 e,f) and OCP (Figure 9- 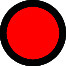) tests.

Figure 11 shows the sample anodized in H_3_PO_4_ + HF (a) without exposure to SBF with magnification 1000x (Figure 2a) and (b) (c) after 96 h of exposure to SBF with magnifications of 1000x and 3000x, respectively.

**Figure 11:**
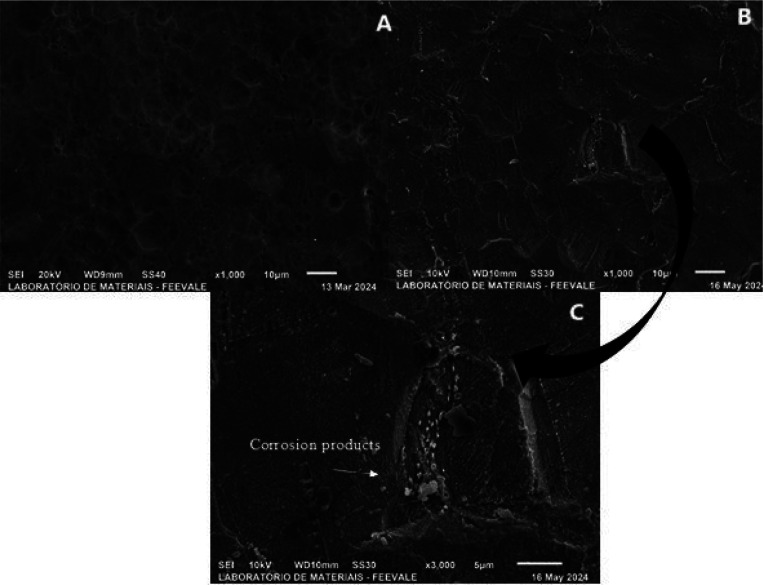
Top-view micrographs of titanium anodized in H_3_PO_4_ + HF (a) before immersion in SBF at 1000× magnification (Figure 4a), and (b, c) after 96 h of immersion in SBF at 1000× and 3000× magnification, respectively.

In Figure 11 (a), it can be noted that the micrograph of titanium anodized in H_3_PO_4_ + HF is different with regard to the equivalent magnification of 1000x when compared to Figure 11 (b). Because of the change in morphology, the pores no longer could be seen due to the corrosion products on the surface that act as a barrier to the permeation of the electrolyte, which corroborates the EIS results in Figure 5 (g,h). When compared to the micrographs of titanium anodized only in H_3_PO_4_, Figure 11 (b) also shows that the corrosion products formed have different morphologies, which is attributed to the presence of HF. This also justifies the difference in the anticorrosive behavior between the H_3_PO_4_ samples with and without HF.

### 3.6 μ-Raman Analysis

To investigate the crystal structure of the oxide layers, μ-Raman spectroscopy analysis was carried out. Considering the satisfactory results for corrosion yielded by the sustainable electrolyte, μ-Raman spectra were obtained for (a) nonanodized and (b) *Psidium guajava*-anodized grade 2 titanium, as reported in Figure 12.

**Figure 12:**
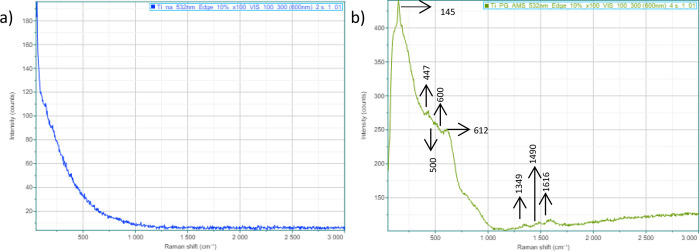
μ-Raman spectra for (a) as-received and (b) *Psidium guajava*-anodized grade 2 titanium.

The intensity of the Raman peak is an indication of the concentration of a compound in the sample and can be used as a quantitative tool to construct curves with different concentrations, obtaining standard spectra. However, this research aims to perform a qualitative analysis to identify the compounds in the samples, considering the peaks with the highest intensity to be indicative of a higher concentration of the substances, mainly TiO_2_, in each sample analyzed.^43,44^

When examining the spectra obtained, no significant peaks are observed in Figure 12 (a). This is because the nonanodized grade 2 titanium sample does not have oxides with a considerable thickness for detection at various points of the μ-Raman spectrum, since oxides formed in air are very thin and lack the properties required to be used in biomaterials.^7^ In contrast, the sample anodized in *Psidium guajava* (Figure 12b) showed an initial peak that is wider than the others, at around 145 cm^–1^. The broadening of the peaks may indicate greater disorder due to the presence of oxygen or a greater number of roto-vibrational movements in the chemical structure.^45^ In addition, three peaks are also observed at around 447, 500, and 612 cm^–1^, which are, according to the literature, characteristic of TiO_2_. According to Aslam et al., (2022),^46^ the peaks at 144.5 and 197.83 cm^–1^ correspond to the presence of Ti–Ti bonds in the octahedral chains, and the peaks observed at 402.62, 524.06, and 642.64 cm^–1^ are generated by Ti–O bonds. This complements the study by Muthuvel et al., (2021),^47^ who obtained Raman peaks at around 139, 162, 365, 498, and 620 cm^–1^ corresponding to the anatase phase of TiO_2_, with the intense peak at 139 cm^–1^ being related to Ti–Ti covalent interactions. Almohammadi et al., (2020)^48^ also reported peaks at 147, 395, 518, and 640 cm^–1^ characteristic of TiO_2_ in the anatase phase.

However, according to Galdos et al., (2017),^49^ the peaks related to the crystalline rutile form are at 447 and 612 cm^–1^. These same peaks were identified in the μ-Raman spectrum in Figure 11 (b). The crystalline structures of TiO_2_, anatase and rutile, stand out for being more stable than the amorphous structures, which indicates a low probability of dissolution of the oxides in body fluids and may justify the better performance observed in the impedance results of the titanium samples anodized in *Psidium guajava*.^50^

The peaks identified at 600 and 1616 cm^–1^ are characteristic of phenolic compounds, especially quercetin.^51^ Furthermore, the 1349 cm^–1^ and 1490 cm^–1^ peaks are related to the bonds containing carbon or O–H vibrations in the phenolic compounds. In the research by Pompeu et al., (2018),^52^ some spectral signs at the 1310–1410 cm^–1^ range were attributed mainly to the O–H deformation vibrations and the C–O stretching of phenolic compounds. Additionally, at 1344 cm^–1^, μ-Raman spectrum responses for C–CH can be seen. The peak at 1490 cm^–1^ obtained in the present study is characteristic of C–C (1498 cm^–1^) or CH_2_ (1487 cm^–1^) bonds.^53^ Both phenolic compounds extracted from *Psidium guajava leaves* (quercetin and ascorbic acid) contain these bonds.^13^

### 3.7 Photocatalytic Activity

Figure 13 below is a photographic image taken after the completion of the photolysis test, where the degradation of the methylene blue dye was analyzed during 150 min of exposure to UV–C light (λ = 280 nm). The tests were performed in triplicate for each of the three Ti samples. The discoloration of the methylene blue dye can be observed with time under UV–C irradiation, as the blue color of the solution gradually decreases.

**Figure 13:**
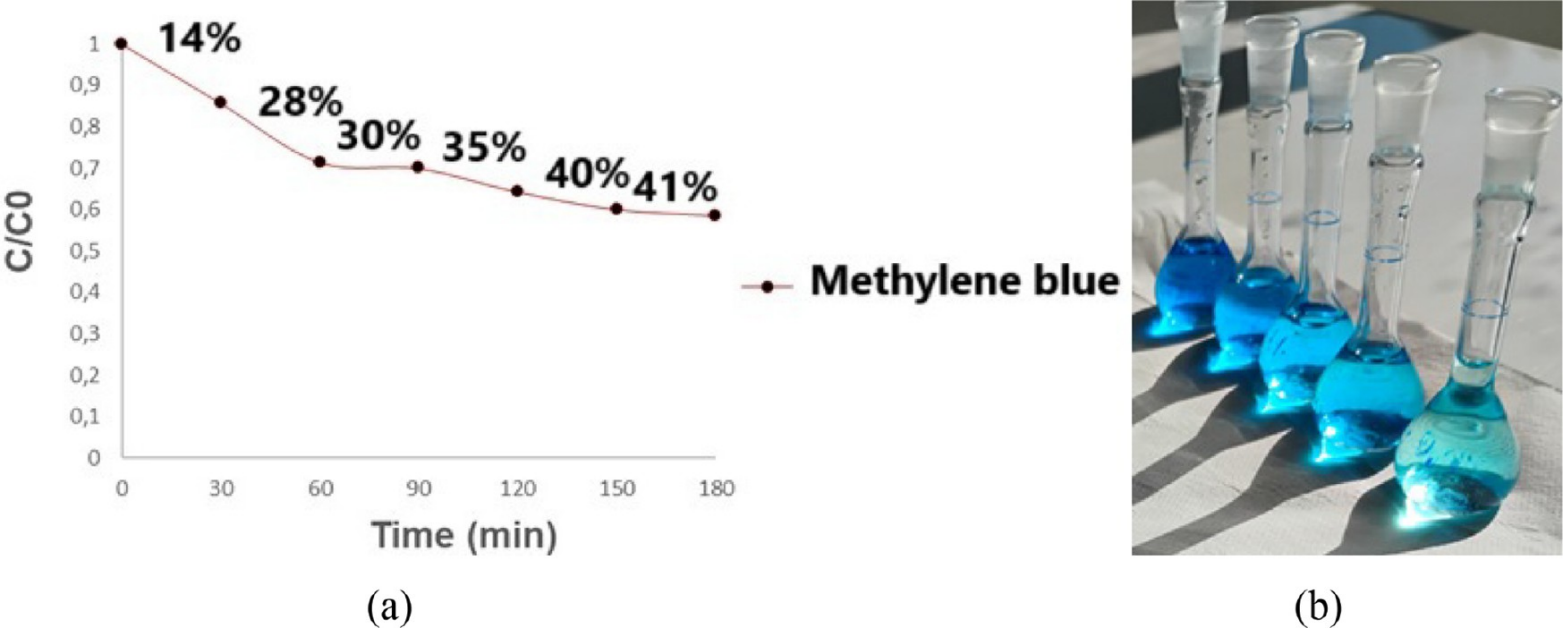
Photolysis test without the presence of the photocatalyst. (a) Photolysis graphs plotted after spectrophotometric analysis. (b) Volumetric flask containing the solution resulting from the photolysis test.

Analyzing the images in Figures 13, and and comparing them, it can be seen that the photolysis test was effective in degrading the methylene blue (MB) dye, showing a discoloration of approximately 40%, respectively. However, when the photocatalysts are supported in solution and irradiated with UV–C light, the catalytic activity increases considerably, practically doubling, reaching approximately 100% when using the titanium photocatalyst anodized in *Psidium guajava*. This occurs because UV light irradiation only has the energy to directly break the bonds of the methylene blue, causing its discoloration. When the photocatalyst is supported in the methylene blue solution, it absorbs light energy and generates oxidizing species that attack the dye, such as hydroxyl radicals (•OH) and superoxide ions (O_2_•^–^), increasing process efficiency.[Fig fig15] [Fig fig14]

**Figure 14:**
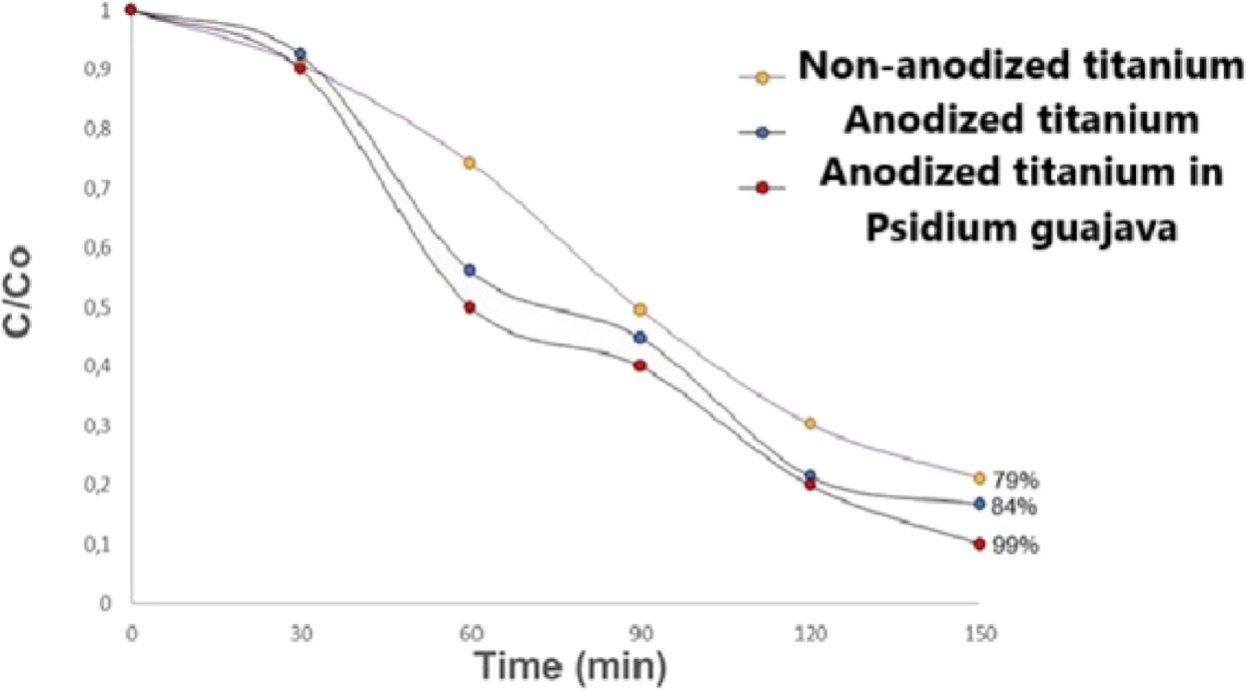
Photocatalytic activity of the semiconductors obtained from the decolorization of methylene blue.

To investigate the photoactivity of the samples in the degradation of methylene blue dye, grade 2 titanium parts, anodized/nonanodized, and anodized with *Psidium guajava*, were used as semiconductors. Figure 16 shows the graphs of the obtained samples. These graphs were plotted after spectrophotometric analysis of the aliquots shown in Figure 15. Analyzing the graphs, it can be observed that all the photocatalysts obtained showed photocatalytic activity in the degradation of methylene blue.

**Figure 15:**
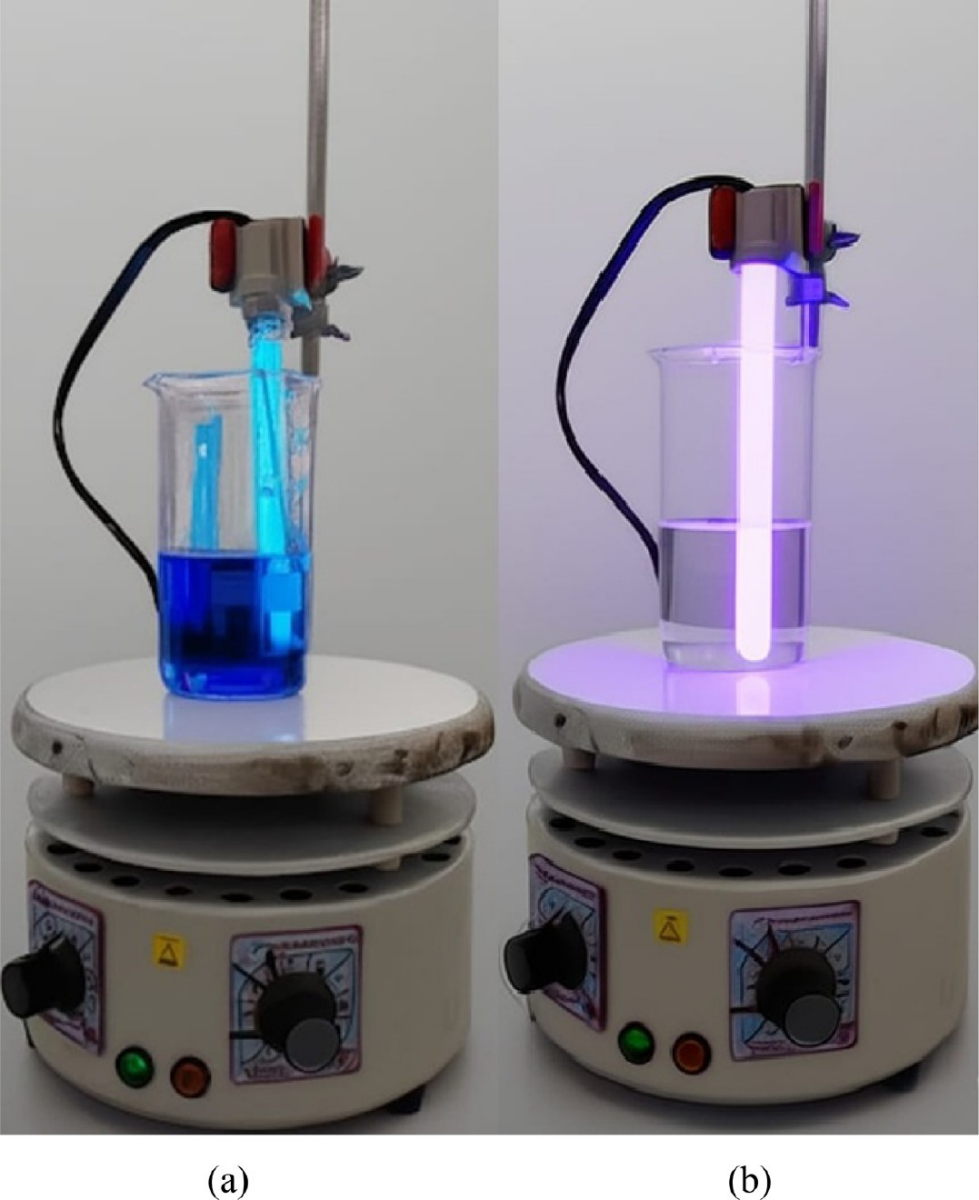
Photocatalytic activity of titanium semiconductor anodized in *Psidium guajava* under UV–C irradiation. (a) At time zero; (b) at 150 min of exposure.

For the nonanodized grade 2 titanium sample, the anatase phase of TiO_2_ was identified, which was confirmed by the peaks presented in section 3.6 of this study. This result was expected, since the anatase phase is proven to be the most photoactive crystalline structure of TiO_2_. The nonanodized grade 2 titanium sample degraded 79% of the methylene blue dye. In its crystalline form, TiO_2_ has a band gap energy of around 3.2 eV, which makes it effective at radiation up to 385 nm,^43,54^ making its use under visible irradiation unfeasible. And in this work, TiO_2_ presented a band gap energy between 3.2 and 3.4. Among the three samples used as photocatalysts, the unanodized grade 2 titanium sample exhibited the lowest photoactivity and the largest bandgap, approximately 3.42 eV (Table 2), which directly impacted the heterogeneous photocatalysis process. This high bandgap value causes rapid recombination of the electron/hole pair, thus limiting the absorption of UV radiation. Another explanation for this lower photoactivity is the semiconductor’s reduced ability to adsorb to the photocatalyst surface. Adsorption is extremely important because it generates a greater number of surface defects, which act as traps for electrons and holes, thus increasing the efficiency of charge separation.^54^

**Table 2:** Band Gap Values of the Synthesized Semiconductors

semiconductors	band gap (eV)
nonanodized titanium	3.42
anodized titanium	3.22
*Psidium guajava*-anodized titanium	3.18

The anodized titanium sample showed a photoactivity of 84%, respectively. The peaks at approximately 139 cm^–1^, 162 cm^–1^, 365 cm^–1^, 498 cm^–1^ and 620 cm^–1^ correspond to the anatase phase of TiO_2_, and at 447 cm^–1^ and 612 cm^–1^ correspond to the rutile phase of TiO_2_, respectively. The increase observed in the photoactivity of this sample is due to the mixture between the anatase/rutile TiO_2_ phases.

The titanium sample anodized in *Psidium guajava* showed the highest photoactivity, with 99% photocatalytic activity in the degradation of methylene blue. The peaks at approximately 139 cm^–1^, 162 cm^–1^, 365 cm^–1^, 498 cm^–1^ and 620 cm^–1^ correspond to the anatase phase of TiO_2_, and at 447 cm^–1^ and 612 cm^–1^ correspond to the rutile phase of TiO_2_, respectively. The increase observed in the photoactivity of this sample is due to the mixture between the anatase/rutile TiO_2_ phases. The presence of this mixture allowed the charge carriers to transition from one crystallographic phase to another. While the electrons move in one direction, the vacancies move in the opposite direction. This phenomenon results in a ″trapping″ of these carriers, hindering the recombination between the electron/hole pair. With the inhibition of the recombination rate, the charge carriers remain available for longer to interact with the molecules adsorbed to the catalyst surface, leading to a greater process efficiency.^40,54,55^

In 2024, Soares et al.,^56^ found that it was possible to successfully produce TiO_2_ and TiO_2_ nanostructures containing tungsten precursors through a microwave-assisted hydrothermal route, and that these nanostructures had good photocatalytic activity, due to the presence of the anatase phase associated with the reduction of the TiO_2_ band gap, which allowed a high degradation of the methyl orange dye in addition to inhibiting the recombination of the electron/hole pair.

Scanlon et al., (2013)^57^ also observed that samples containing anatase/rutile showed a higher photocatalytic activity than the individual polymorphs. According to m, one explanation for this higher photocatalytic activity observed in samples containing the anatase/rutile mixture is due to the position of the valence and conduction bands of TiO_2_. The anatase polymorph has a band gap of 3.2 eV and the rutile phase of 3.03, respectively.^43^ The flat band potential of the anatase phase is approximately 0.2 eV more negative than that of the rutile phase. Indicating that the conduction band of the anatase phase is located 0.2 eV above the rutile phase. This alignment between the bands favors photocatalytic activity, the transfer of photogenerated electrons and holes from the rutile phase to the anatase phase.

Montanhera et al.*,* (2016)^58^ also observed this phenomenon of increased photoactivity of their samples when a mixture of the anatase and rutile phases of TiO_2_ was present. In their study, the authors synthesized TiO_2_ by an alternative route little explored in the literature, which consists of adding titanium oxysulfate and hydrogen peroxide in aqueous solution. The results obtained showed that there is an increase in photocatalytic efficiency for samples containing a small percentage of rutile compared to samples containing a pure anatase phase.

The lowest band gap value was observed for the *Psidium guajava*-anodized Ti grade 2 (Table 2) semiconductor, which also explains its much better catalytic activity compared to other samples. This reduction in the band gap probably occurred because rutile is a semiconductor that has a direct band gap, while anatase is indirect, which causes the photogenerated carriers to recombine more quickly than when only the anatase phase is present.^40,58,59^

Another reason for the better photoactivity (99%) shown by these samples is the presence of phenolic groups, more specifically quercetin, with characteristic peaks at 600 and 1616 cm^–1^.^51^ Therefore, the points observed in quantity in the micrograph of the *Psidium guajava*-anodized Ti grade 2 (Table 2) sample are possibly quercetin nanoparticles present in the plant extract, which exert an oxidizing effect. It is known that *Psidium guajava* leaves have phenolic compounds that help in the generation of versatile effects, both reducing and oxidizing.^60,61^ To prove this theory, the aqueous extracts of *Psidium guaiava* leaves were characterized in terms of chlorophyll A and B, carotenoids and total phenolic compounds. The average levels of chlorophyll A and B were 0.397 mg/g and 0.470 mg/g, respectively. The average levels of carotenoids were 0.160 mg/g. The levels of phenolic compounds were fully determined by the Folin-Ciocalteau spectrophotometric method and expressed as equivalents to pyrogallol g/100 mL of extractive solution. Thus, an average of 0.1895% was found for extractive solutions obtained at room temperature and 0.2336% for those obtained by infusion. After anodizing, these levels decreased to 0.1493% and 0.1963%, respectively, proving that the phenolic compounds acted as oxidizing agents in the anodizing process.^60^

This oxidizing potential of the quercetin phenolic group occurs due to the formation of hydroxyl radicals (*OH), which have a high manipulation capacity. Because the grade 2 titanium semiconductor anodized in *Psidium guajava* absorbs UV light, it promotes the attraction of electrons from the valence band to the conduction band. This electronic technology generates electron/hole pairs on the surface of the semiconductor, which react with oxygen (O_2_) and water (H_2_O) molecules, forming oxidizing radicals (hydroxyl). In turn, these radicals promote the manipulation of organic compounds, the trapping of excited electrons and the prevention of recombination of e^–^/h^+^ pairs. The decrease in the chances of recombination allows for greater charge separation, generating more active sites for the adsorption of reactant molecules, increasing the number of surface defects, and extending and facilitating the transfer of electron/hole pairs, thus promoting the greater photocatalytic activity of this sample.^60^

The Table 2 presents the band gap values of the samples of nonanodized titanium, anodized titanium and titanium anodized in *Psidium guajava.*

Figure 16 shows the band gap energy graphs, which were plotted according to the Kubelka–Munk method and formula E­(eV) = 1240/λ (nm), after measurements performed by diffuse reflectance spectroscopy. It can be observed that the lowest band gap value was obtained by the *Psidium guajava* anodized titanium semiconductor, 3.18 eV, respectively. This corroborates the greater photocatalytic activity that this sample demonstrated in the degradation of the methylene blue dye.

**Figure 16:**
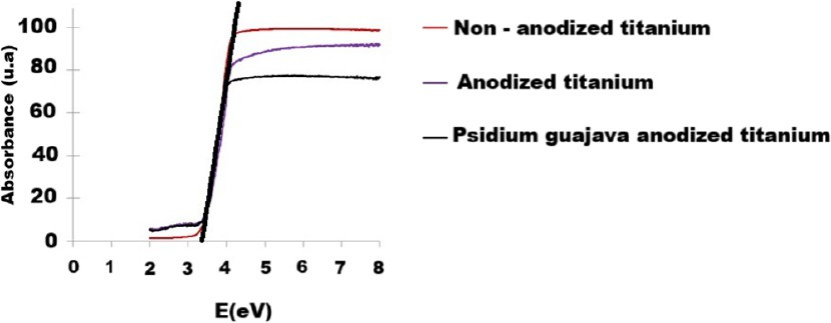
DRS analysis for plotting graphs and determining band gap energy.

## Conclusions

The grade 2 titanium used for the study was annealed, presenting grains of approximately 10 μm. The pickling process in HNO_3_ + HF formed cracks on the titanium surface as well as its passivation. The oxides formed in both H_3_PO_4_ and *Psidium guajava* are quite thin and presented a morphology similar to that of the pickled sample. The pickling process increased the surface roughness of the titanium, practically doubling the value, due to the chemical attack with HNO_3_ + HF. The roughness values were similar for the samples anodized in H_3_PO_4_ and in *Psidium guajava*, and showed little variation compared to the pickled sample, indicating that the oxides formed are quite thin. The addition of HF in the anodization with H_3_PO_4_ resulted in lower roughness among the anodized samples, which leads to the conclusion that the oxide filled the surface imperfections, due to the formation of porous oxide, and this porosity is not detected by the profilometry technique. Regarding the visual chromatic analyses for the anodized samples, it was found that the H_3_PO_4_ and *Psidium guajava* samples obtained a similar golden coloration, characteristic of low potential anodization. For the sample anodized in H_3_PO_4_ + HF electrolyte, the yellowish coloration was lighter than the others. For the EIE tests, it was observed that in the pickled titanium sample, at all immersion times in SBF, metal passivation occurs during the pickling process, associated with the permeation of the electrolyte through the cracks in the passive layer formed during pickling, which possibly contributed to the acceleration of the corrosion process. In the titanium anodized in *Psidium guajava* and in H_3_PO_4_ + HF, the impedance test showed the formation of corrosion products with temporary protection against electrolyte permeation. It was observed that the *Psidium guajava* sample presented better electrochemical performance, which was associated with the oxidizing effect due to the phenolic compounds present in the electrolyte. The OCP tests confirmed the results obtained for EIE, with better anticorrosive performance being obtained for the samples anodized in *Psidium guajava*. The lowest band gap value was observed for the Ti grade 2 semiconductor anodized in *Psidium guajava*, which gave it greater catalytic activity, compared to other samples. This reduction in the band gap probably occurred because rutile is a semiconductor with a direct band gap, while anatase is indirect, which causes the photogenerated carriers to recombine more quickly than when only the anatase phase is present.
